# Severe Hypocalcemia Dependent on Fluconazole in a Newborn With Barakat Syndrome

**DOI:** 10.1155/crpe/1394105

**Published:** 2025-07-10

**Authors:** Dorota Roztoczyńska, Anna Wędrychowicz, Magdalena Ossowska, Aleksandra Furtak, Paulina Miśkiewicz-Stanek, Ewelina Preizner-Rzucidło, Joanna Kwinta-Rybicka, Mateusz Jagła, Jerzy B. Starzyk

**Affiliations:** ^1^Department of Pediatric and Adolescent Endocrinology, Department of Pediatrics, Institute of Pediatrics, Collegium Medicum, Jagiellonian University, Krakow, Poland; ^2^Department of Genetics, Institute of Pediatrics, Collegium Medicum, Jagiellonian University, Krakow, Poland; ^3^Department of Pediatric Nephrology and Hypertension, Institute of Pediatrics, Collegium Medicum, Jagiellonian University, Krakow, Poland; ^4^Department of Neonatology and Intensive Care, Department of Pediatrics, Institute of Pediatrics, Collegium Medicum, Jagiellonian University, Krakow, Poland

**Keywords:** hearing loss, hypoparathyroidism, kidney diseases, neonatal hypocalcemia

## Abstract

We present a case of a newborn with Barakat syndrome, characterized by congenital hypoparathyroidism, severe hypocalcemia, recurrent urinary tract infections (UTIs), and congenital candidiasis, which is an atypical feature for this syndrome. The patient, born at term, exhibited dysmorphia, hearing loss, and renal dysfunction. Genetic testing revealed a novel, de novo *GATA3* variant. Fluconazole, introduced to treat recurrent UTIs and congenital candidiasis, unexpectedly played a crucial role in normalizing calcium levels. This effect may be attributed to fluconazole's influence on the metabolism of vitamin D, potentially enhancing calcium absorption and reabsorption. The normalization of calcium levels in this patient emphasizes the complex interplay between antifungal therapy and calcium homeostasis, particularly in patients with congenital hypoparathyroidism. This case highlights the importance of genetic testing in diagnosing neonatal hypocalcemia and illustrates the potential for fluconazole to impact calcium metabolism in Barakat syndrome. A multidisciplinary approach, including immunological, nephrological, and otolaryngological evaluations, is essential for comprehensive long-term care.

**Summary**

• Early genetic diagnosis of neonatal hypocalcemia allows for the prediction of disease course and implementation of appropriate treatment.
• In this case, severe hypocalcemia was temporally associated with fluconazole treatment, suggesting a possible drug-related effect.
• In cases of multisystemic and atypical disorders, a broad spectrum of tests significantly increases the chance of accurate diagnosis.
• The patient requires extended immunological, nephrological, and otolaryngological diagnostics as well as multidisciplinary long-term care.

## 1. Introduction

Hypocalcemia is defined as total serum calcium < 1.75 mmol/L in preterm neonates, < 2 mmol/L in term neonates, and < 2.2 mmol/L in children [1]. Early-onset hypocalcemia within the first 72 h of life is caused by prematurity, small for gestational age (SGA), intrauterine growth restriction (IUGR), birth asphyxia, maternal diabetes, toxemia, bicarbonate administration, intralipid, or citrate blood. Late-onset hypocalcemia after 72 h is often due to a high phosphate content in the diet, vitamin D deficiency, hypomagnesemia, hypoparathyroidism or parathyroid hormone resistance, and kidney and liver diseases. Congenital hypoparathyroidism must be ruled out for persistent hypocalcemia in newborns. The condition can be isolated (sporadic or familial with autosomal dominant (AD), autosomal recessive (AR), or X-linked recessive (XR) inheritance) or associated with mutations affecting parathyroid development genes, such as *GCM2* (6p24.2) and *SOX3* (2p25.3, Xq27.1), or PTH synthesis and secretion genes (prepro-PTH—11p15Congenital hypoparathyroidism is often a part of syndromic conditions such as DiGeorge syndrome (del.22q), HDR syndrome (AD), Kenny–Caffey syndrome, Sanjad–Sakati syndrome (1q42-q43), or mitochondrial DNA-related conditions such as Kearns–Sayre syndrome and MELAS [2]. Genetic testing is crucial for accurate diagnosis and treatment of neonatal hypocalcemia.

## 2. Case Report

**Figure 1:**
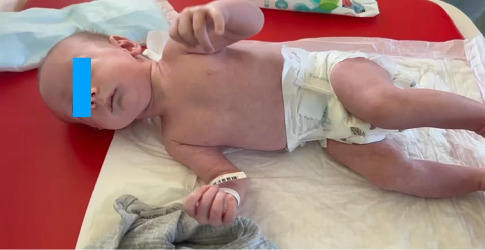
Patient with facial dysmorphia.

The boy was born at term (40 weeks) from the first pregnancy, with a body mass of 3180 g, and an Apgar score of 9/10. Postnatally, he presented with oral thrush, limited hip abduction, heel-positioned feet, facial dysmorphia: retruded and asymmetric jaw, broad forehead, lack of eyebrows, almond-shaped prominent eyes, slightly thinner upper lip, subtly higher columella, and slightly lower ears (Figure 1).

Neonatal hearing screening revealed no response, necessitating follow-up. Laboratory tests revealed elevated levels of inflammatory markers, hypocalcemia, and hypomagnesemia (Table 1). After correcting the electrolyte imbalances, the patient was discharged. On the 10th day of life, he was diagnosed with a urinary tract infection (UTI) and admitted to the Neonatal Intensive Care Unit of the University Children's Hospital in Krakow, where hypocalcemia, increased inflammatory marker values, and signs of acute kidney injury (increased echogenicity of renal pyramids on ultrasound, leukocyturia, erythrocyturia, proteinuria, and elevated serum creatinine) were observed. Blood and urine cultures revealed *Escherichia coli*, and throat and stool cultures showed yeast-like fungi. At 20 days of life, an endocrinologist diagnosed hypocalcemia (Ca: 1.57 mmol/L; normal: 2.17–2.44 mmol/L), magnesium level within the normal range but close to the lower limit (Mg: 0.50 mmol/L; normal: 0.48–1.05 mmol/L), hyperphosphatemia (P: 2.99 mmol/L; normal: 1.50–2.10 mmol/L), and congenital hypoparathyroidism (PTH < 4.6 pg/mL; normal: 4.8–40 pg/mL). The serum concentration of 25-hydroxyvitamin D [25(OH)D] was 24.6 ng/mL (reference range: 30–50 ng/mL). The patient was prescribed magnesium aspartate, calcium carbonate, alfacalcidol, and nystatin. The ACTH, cortisol, TSH, FT4, IGF1, and maternal calcium levels were normal. Genetic tests excluded *CASR* gene mutations and copy number changes, including 22q11 deletion, relevant to the child's dominant symptoms. He was transferred to the pediatric endocrinology unit (01 January 29, 2024), where calcium carbonate, magnesium preparations, and alfacalcidol were increased, fluconazole was introduced, and a low-phosphate diet (Renastart) was implemented, achieving normocalcemia (Ca: 2.11 mmol/L). Fluconazole was discontinued following UTI recurrence and nephrological consultation. Despite the increase in calcium carbonate (8 × 100 mg) and calcitriol (Detriol max. 2 μg/d) doses, his calcium levels dropped to Ca.c.: −1.3 mmol/L. The reintroduction of fluconazole normalized the electrolyte levels (Table 1).

Immunological studies showed no abnormalities, except for T cell activation during UTI. Blood samples were collected for whole exome sequencing (WES). The child was discharged in a good general condition.

In the 4th month of life, the child experienced recurrence of UTI. WES revealed a new nonsense variant of potentially pathogenic significance in *GATA3* gene (NM_001002295.2(GATA3): c.1063delC (p.Leu355fs)), meets PVS1, PM2, and PP5 criteria according to ACMG. Variant causes a frameshift change involving the alteration of a conserved nucleotide. The variant was absent in control chromosomes in GnomAD project. Variant has been reported in Lovd as Pathogenic. Additionally family segregation analysis confirmed that the variant occured *de novo*. *GATA3* gene pathogenic variants are associated with Barakat syndrome, which is characterized by hypoparathyroidism, deafness, and kidney abnormalities. The child's symptoms matched those of the identified variant.

## 3. Discussion

*GATA3*, located on chromosome 10 at region 10p14, plays a key role in embryogenesis, including the development of the parathyroid glands, auditory system, kidneys, thymus, liver, adrenal glands, and central nervous system. It is also an important transcription factor regulating the immune system—primarily the differentiation of T helper (Th) lymphocytes into the Th2 subtype and its expression in thymus development [2]. Pathogenic variants of GATA3 may disrupt the function of various protein domains, especially the zinc finger domains (ZnF1 and ZnF2), which are essential for DNA binding and interaction with other proteins.

The HDR syndrome was first described by Barakat and colleagues, who analyzed 180 cases: hypoparathyroidism (“H”) was found in 93.3% of the patients, sensorineural deafness (“D”) in 96.7%, and renal abnormalities (“R”) in 72.2% [3]. None of the patients exhibited clinical or biochemical features of immunodeficiency. Immunological disturbances in some patients with 10p deletions are most likely the result of damage to other genes in this region. Similarly, facial dysmorphism, growth disturbances, and developmental delay observed in individuals with larger 10p deletions were not present in patients with point mutations (SNVs) in the GATA3 gene, which also indicates the involvement of other genes [4]. WES in our patient also revealed the presence of a variant in the LAT gene, whose protein product is associated with AR early-onset immunodeficiency with autoimmunity [5]. In our case, aside from T lymphocyte activation during UTIs, no other abnormalities were found in immunological tests. However, it should be emphasized that congenital candidiasis is not a typical symptom of Barakat syndrome. The presence of candidiasis in this context may suggest a subtle defect in T lymphocyte function, particularly in the IL-17 pathway, without overt features of immunodeficiency [6, 7]. GATA3 is a key transcription factor not only for Th2 cells but also for ILC2 cells—responsible for mucosal defense. It regulates immune homeostasis and dose dependently influences T lymphocyte maturation and cytokine production. Dysfunction of this factor may lead to weakened immune response at the mucosal level, increasing susceptibility to fungal infections such as candidiasis [8].

Congenital immunodeficiencies with candidiasis, T lymphocyte dysfunction, and IL-17 deficiency have been described in the context of pathogenic variants in genes such as STAT3, CARD9, DOCK8, ACT1, IL-17RC, IL-17RA, and IL-17F—where fluconazole is the first-line treatment [9]. Other antifungal drugs, such as itraconazole, posaconazole, or voriconazole, are significantly more toxic due to stronger inhibition of cytochrome P450 isoenzymes in the liver.

Normalization of calcium levels after fluconazole treatment could have resulted from improved vitamin D metabolism rather than from mechanisms directly related to immunodeficiency, which was not confirmed in our patient.

**Figure 2:**
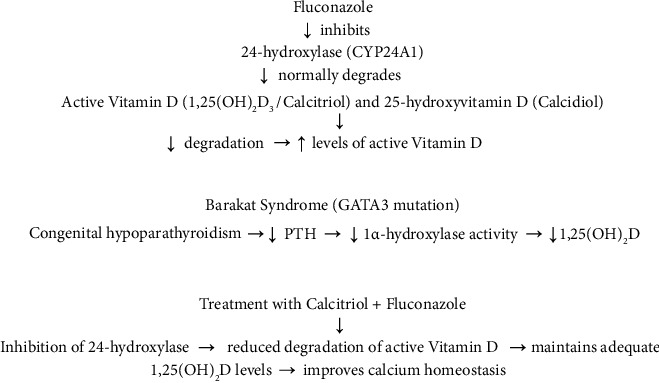
Mechanism of the effect of calcitriol and fluconazole treatment on vitamin D and calcium metabolism in Barakat syndrome (GATA3 mutation).

Fluconazole, a widely used azole antifungal drug, inhibits the activity of several cytochrome P450 enzymes, including 24-hydroxylase (CYP24A1), which is responsible for the catabolism of the active form of vitamin D, 1,25-dihydroxyvitamin D3 (calcitriol), and its precursor, 25-hydroxyvitamin D (calcidiol), into inactive metabolites. By inhibiting this enzyme, fluconazole reduces calcitriol degradation, resulting in increased levels of active vitamin D in the body [10, 11]. In addition, azoles may also decrease the activity of 1α-hydroxylase (CYP27B1), which theoretically could limit calcitriol synthesis. However, in patients with reduced production of 1,25(OH)_2_D, such as those with congenital hypoparathyroidism (e.g., Barakat syndrome), inhibition of calcitriol degradation may have therapeutic significance. By limiting the catabolism of active vitamin D, fluconazole helps maintain higher calcitriol concentrations, which in turn improves calcium regulation and its absorption in the gastrointestinal tract (Figure 2).

From a clinical point of view, this property of fluconazole can constitute an important supportive adjunct in the treatment of patients with vitamin D deficiency and hypocalcemia secondary to hypoparathyroidism, especially when standard supplementation with the active form of vitamin D (calcitriol) is insufficient.

Typical features of HDR syndrome have also been described in patients with 10p deletions, who present clinical features of DiGeorge syndrome (DGS). Studies of patients with 10p deletions identified two nonoverlapping regions responsible for specific phenotypes: the critical region of DiGeorge syndrome 2, located in 10p13-14, and the HDR syndrome region, located telomerically at 10p14–10pter [4, 12]. However, can we state that our patient, in addition to HDR, shows features of DiGeorge syndrome such as dysmorphia and cellular immunodeficiency? Previous studies have shown that pathogenic point variants (SNVs) in the GATA3 gene do not cause facial dysmorphism, which is considered a differentiating feature in the genetic diagnosis of congenital hypoparathyroidism [13]. Besides immunodeficiency, dysmorphic features and hearing loss in the patient require further diagnostics.

## Figures and Tables

**Table 1:** Treatment of hypocalcemia.

Age in weeks	1 week (29.12.2023)	5 weeks (29.01.2024)	6 weeks (12.02.2024)	8 weeks (18.02.2024)	9 weeks (26.02.2024)
Treatment	Calcium gluconate (?) 20% MgSO_4_ i.v. No dose information	Alfacalcidol 0.5 μg/d, calcium carbonate 4 × 200 mg/d, magnesium aspartate 3 × 300 mg/d	Alfacalcidol 0.75 μg/d, calcium carbonate 4 × 200 mg/d, magnesium aspartate 4 × 300 mg/d, fluconazole 4 × 5 mg/d, Renastart diet 2 × 60 mL/d	Calcitriol 2 × 0.5 μg, calcium carbonate 5 × 200 mg, magnesium aspartate 4 × 300 mg/d, Renastart diet 3 × 60 mL/d	Calcitriol 2 × 0.5 μg, calcium carbonate 14 × 100 mg, magnesium aspartate 6 × 300 mg/d, fluconazole 4 × 5 mg/d, hydrochlorothiazide 2 × 2 mg, Renastart diet 5 × 80 mL/d
Ca.c. (mmol/L) normal	1.87 (2.15–2.55)	1.56 (2.39–3.05)	2.11 (2.39–3.05)	1.43 (2.39–3.05)	2.00 (2.39–3.05)
P (mmol/L) normal	2.19 (0.81–1.45)	3.23 (1.5–2.6)	3.40 (1.5–2.6)	3.29 (1.5–2.6)	2.86 (1.5–2.6)
Mg (mmol/L) normal	0.61 (0.66–1.07)	0.67 (0.48–1.05)	—	0.57 (0.48–1.05)	0.84 (0.48–1.05)
Creatinine (μmol/L) normal	78 (59–104)	56.8 (13.3–23.9)	44.4 (13.3–23.9)	36.1 (13.3–23.9)	41.2 (13.3–23.9)

## Data Availability

The data that support the findings of this study are available from the corresponding author upon reasonable request.
